# 4411 Identifying Environmental Barriers to Participation for Community-Dwelling Adults with Stroke: A Descriptive Pilot Study

**DOI:** 10.1017/cts.2020.398

**Published:** 2020-07-29

**Authors:** Anna J Neff, Stephen Lau, Alex Wing-Kai Wong, Carolyn Baum

**Affiliations:** 1 Washington University in St. Louis, Institute of Clinical and Translational Sciences; 2 Washington University School of Medicine

## Abstract

OBJECTIVES/GOALS: The purpose of this study is to identify and quantitatively describe environmental barriers to community engagement and activity participation for adults with stroke and low income. Repeated electronic surveys collected in real time will reduce recall bias and improve characterization of barriers. METHODS/STUDY POPULATION: 20-30 community-dwelling adults with stroke and low income will be recruited for this pilot study. Inclusion criteria: > 1 month post stroke and evidence that they have the vision, literacy, and cognitive capacities to answer survey questions on a smart device. Exclusion criteria: severe aphasia, severe mental illness or substance abuse within 3 months, and ataxia. Participants will complete standardized assessments of daily activities, engagement in and perceptions about community activities, social support, and perceived environmental barriers. Participants then complete four surveys per day for 14 days using an app on an iPod Touch, reporting activities attempted and barriers encountered. RESULTS/ANTICIPATED RESULTS: This is the first study of this kind and is a work in progress. We anticipate that the environmental barriers reported will include physical (e.g. built structures, climate, and natural terrain), social (e.g. support or lack thereof; stigma), political (e.g. access to transportation; healthcare services), and technological barriers (e.g. difficulties with personal equipment and/or technologies such as elevators, ticket kiosks, etc.). DISCUSSION/SIGNIFICANCE OF IMPACT: An increased understanding of the barriers facing community-dwelling adults with stroke and low income will facilitate the development of culturally-appropriate and more accessible self-management programs to help this population re-engage in their communities and return to pre-stroke activities.

